# Treatment of cytomegalovirus anterior segment infection with intravitreal injection of ganciclovir in adjunction with or without oral valganciclovir: a long-term results

**DOI:** 10.1038/s41598-021-82637-y

**Published:** 2021-02-04

**Authors:** Yu-Chun Cheng, Eugene Yu-Chuan Kang, Yih-Shiou Hwang, Ching-Hsi Hsiao

**Affiliations:** 1 Department of Ophthalmology, Chang Gung Memorial Hospital, Linkou, 5 Fu-Hsin Rd, Kweishan 333, Taoyuan, Taiwan; 2 College of Medicine, Chang Gung University, Taoyuan, Taiwan

**Keywords:** Diseases, Medical research, Molecular medicine

## Abstract

We evaluated the therapeutic outcome of intravitreal injection (IVI) of ganciclovir with/without oral valganciclovir for cytomegalovirus (CMV) anterior segment infection. We enrolled 61 patients (61 eyes) with PCR-proven CMV anterior segment infection. IVI of ganciclovir (2 mg/0.05 mL) was given as a loading dose; subsequent use of oral valganciclovir (900 mg twice daily) was determined according to the severity of anterior chamber inflammation after injection. All eyes had IVI of ganciclovir, and 53 patients received oral valganciclovir as adjunctive therapy with a mean duration of 1.9 months to achieve disease remission. Repeated diagnostic aqueous taps were performed in 37 eyes with suspected recurrence, and CMV DNA was positive in 24 eyes. This therapeutic strategy afforded a median 50% recurrence-free survival time of 47.0 ± 8.12 months. The patients’ mean best corrected visual acuity, intraocular pressure and corneal endothelial cell counts stabilized or improved. Corneal transplantation before CMV infection diagnosis was identified as an independent risk factor for recurrence (hazard ratio 6.81, 95% confidence interval 1.21–38.23, *P* = 0.029). In patients with CMV anterior segment infection, the relative short-term therapeutic strategy, IVI of ganciclovir in adjunction with/without oral valganciclovir, effectively achieved a median recurrence-free survival time of nearly 4 years.

## Introduction

Cytomegalovirus (CMV), a double-stranded DNA virus from the herpes virus family, has been clinically identified as a new causative agent of anterior segment infection manifesting as either corneal endotheliitis or anterior uveitis in immunocompetent patients^1–3^. Typical clinical findings of CMV anterior segment infection include elevated intraocular pressure (IOP), corneal edema, coin-shaped keratic precipitates (KP), corneal endothelial cell loss, iris atrophy, and anterior chamber inflammation^4,5^. CMV infection has been identified in patients diagnosed with idiopathic corneal endotheliitis, Posner-Schlossman syndrome, and Fuchs heterochromic iridocyclitis, all of which were previously considered idiopathic^6,7^.

Early initiation and proper maintenance of ganciclovir use interrupts CMV activity. Systemic administration, intravitreal injection or implant, and topical use of ganciclovir, as well as oral valganciclovir, can achieve remission of CMV infection^1,8–10^. However, despite successful inflammation control, the recurrence rate is high after treatment cessation, posing a substantial threat of permanent damage to the trabecular meshwork and corneal endothelium^6,8^. Chronic prophylaxis with oral or topical antiviral agents can prevent CMV reactivation in patients with anterior segment infection^8,11^. However, long-term anti-CMV medication use is costly and may be associated with side effects and resistant strain emergence. Previously, we reported a case series of six patients with CMV anterior uveitis who received intravitreal injection (IVI) of ganciclovir as a loading dose with or without adjunctive oral valganciclovir, and had satisfactory inflammation control without recurrence in a mean follow-up period of 14.7 months in all patients^12^. To determine the long-term therapeutic effect of this treatment strategy for CMV anterior segment infection, we extended our previous study in duration and scale. The CMV infection recurrence and its associated risk factors, IOP change, best-corrected visual acuity (BCVA) change, and endothelial cell loss over time were all evaluated.

## Results

Sixty-four eyes of 61 patients diagnosed with CMV anterior segment infection were examined during the study period. For three patients who had bilateral involvement, we picked up the right eye of two patients with symmetric clinical courses in both eyes, and the eye having recurrence of the third patient for analysis. In total, 61 eyes were included. The demographic data and initial clinical manifestations of the patients are listed in Table 1. The mean age at diagnosis was 54.1 ± 11.5 years. Forty-eight patients (78.7%) were male. The mean follow-up period after diagnosis was 41.0 ± 27.1 months.

**Table 1:** Demographic data and initial clinical manifestation of patients with CMV anterior segment infection.

Characteristics	
Number of patients and diseased eyes, no	61
Age at diagnosis, years^a^	54.1 ± 11.5
Male, no	48 (78.7%)
Mean follow up period, months^a^	41.0 ± 27.1
History of glaucoma surgery, no	6 (9.8%)
History of corneal transplantation, no	7 (11.5%)
History of cataract surgery, no	20 (32.8%)
Mean BCVA in the diseased eye at diagnosis, logMAR^a^	0.75 ± 0.84
Mean IOP in the diseased eye at diagnosis, mmHg^a^	24.0 ± 9.2
Mean ECD in the diseased eye at diagnosis, cells/mm^2a^	1751.7 ± 620.7
Mean ECD in the healthy eye at diagnosis, cells/mm^2a^	2544.1 ± 450.9
Corneal endothelial cell loss in the diseased eye at diagnosis	31.2%

*No.* number, *BCVA* best corrected visual acuity, *IOP* intraocular pressure, *ECD* endothelial cell density.

^a^Demonstrated as mean ± standard error.

Prior to confirmation of CMV infection, all patients received topical IOP-lowering medication, and 98.4% had topical corticosteroid use. Antiviral agents for presumed herpetic infection were used in eight eyes (13.1%), glaucoma surgery for IOP control was performed in six eyes (9.8%), and corneal transplantation was performed in seven eyes (11.5%).

Corneal edema, characteristic coin-shaped KP, and anterior chamber inflammation were found in 52.5%, 37.7%, and 63.9% of all patients, respectively. Twenty eyes (32.8%) were pseudophakic. The mean logMAR BCVA at diagnosis was 0.75 ± 0.84, and the mean IOP at diagnosis was 24.0 ± 9.2 mmHg. Although all the patients received antiglaucoma agents, 30 eyes (49.1%) had elevated IOP (> 22 mmHg). The mean endothelial cell density (ECD) was 1751.7 ± 620.7 and 2544.1 ± 450.9 cells/mm^2^ in the diseased and healthy eyes, respectively. The mean ECD was significantly lower in diseased eyes than in healthy eyes (*P* < 0.001). Corneal endothelial cell loss was 31.2% in the diseased eyes. All examined eyes were positive for CMV DNA, with a mean CMV PCR viral load of 3.96 ± 9.00 × 10^7^ copies/mL. Seven eyes (11.5%) had concomitant positive Epstein–Barr virus (EBV) DNA in the aqueous humor. All eyes were treated with intravitreal ganciclovir injection, and 53 patients received oral valganciclovir as adjunctive therapy, with a mean duration of 1.9 months. Treatment success and quiescence of anterior chamber inflammation were achieved in all patients. None of the patients had complications related to intravitreal ganciclovir injection or oral valganciclovir.

**Figure 1:**
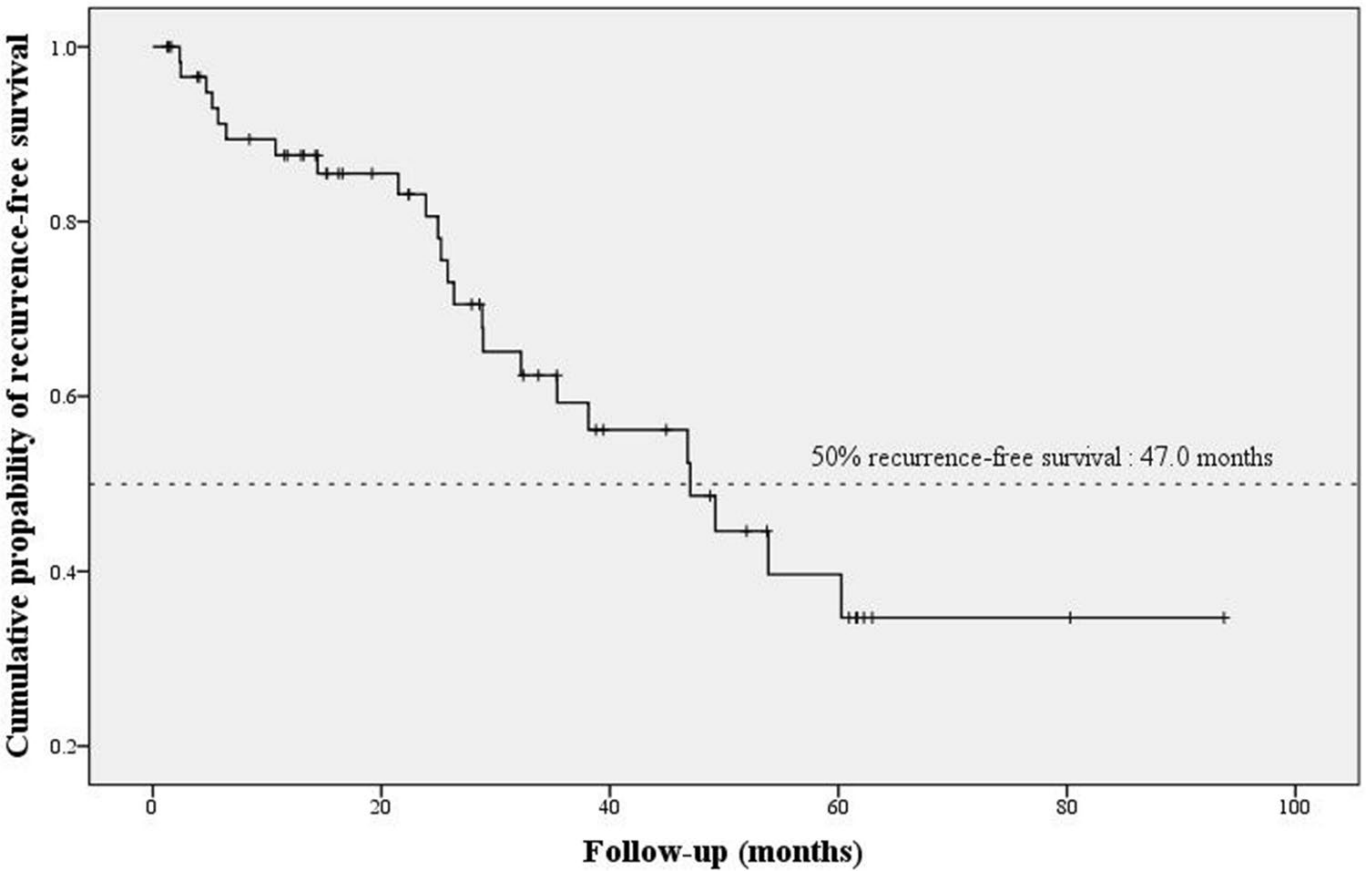
Kaplan–Meier survival curve plotted mean survival time 47.0 months at which 50% of patients were recurrence free.

Repeated diagnostic aqueous taps were performed in 37 eyes, and CMV DNA was tested positive in 24 eyes. Regarding the primary outcome of this study, the time to molecular recurrence was documented by Kaplan–Meier survival analysis in Fig. 1. The median 50% recurrence-free survival time, defined as the duration during which 50% of the patients were free of recurrence of CMV anterior segment infection after intravitreal injection of ganciclovir, was 47.0 ± 8.12 months.

There was no significant difference in initial viral titers between the eyes without recurrence and those with recurrence (3.08 ± 8.70 × 10^7^ versus 6.24 ± 10.00 × 10^7^ copies/mL, *P* = 0.411). We examined demographic data, medical treatment before diagnosis, and clinical presentation at diagnosis to identify risk factors for molecular recurrence of CMV anterior segment infection; the results are listed in Table 2. Multivariate analysis identified corneal transplantation before diagnosis as an independent risk factor (hazard ratio 6.81, 95% confidence interval 1.21–38.23, *P* = 0.029) for molecular recurrence after treatment with intravitreal ganciclovir injection and adjunction of oral valganciclovir.

**Table 2:** Risk factors associated with molecular recurrence.

Variable	Univariate analysis		Multivariate analysis	
	HR (95% CI)	*P*	HR (95% CI)	*P*
**Demographics**				
Age, year	1.04 (0.99–1.09)	0.139	1.01 (0.96–1.07)	0.526
Male	1.43 (0.42–4.92)	0.572		
**Prior to diagnosis**				
Topical antiviral use	0.47 (0.09–2.55)	0.381		
Glaucoma surgery	0.75 (0.13–4.45)	0.752		
Cataract surgery	2.63 (0.88–7.90)	0.084	1.44 (0.39–5.35)	0.587
Corneal transplant	8.75 (1.67–45.95)	0.010	6.81 (1.21–38.23)	0.029
**At diagnosis**				
Cornea edema	1.33 (0.47–3.74)	0.593		
Coin shaped KP	1.32 (0.46–3.79)	0.607		
AC inflammation	1.22 (0.41–3.58)	0.721		
IOP, mmHg	1.00 (0.95–1.07)	0.858		

*HR* hazard ratio, *CI* confidence interval, *KP* keratic precipitates, *IOP* intraocular pressure, *AC* anterior chamber.

**Figure 2:**
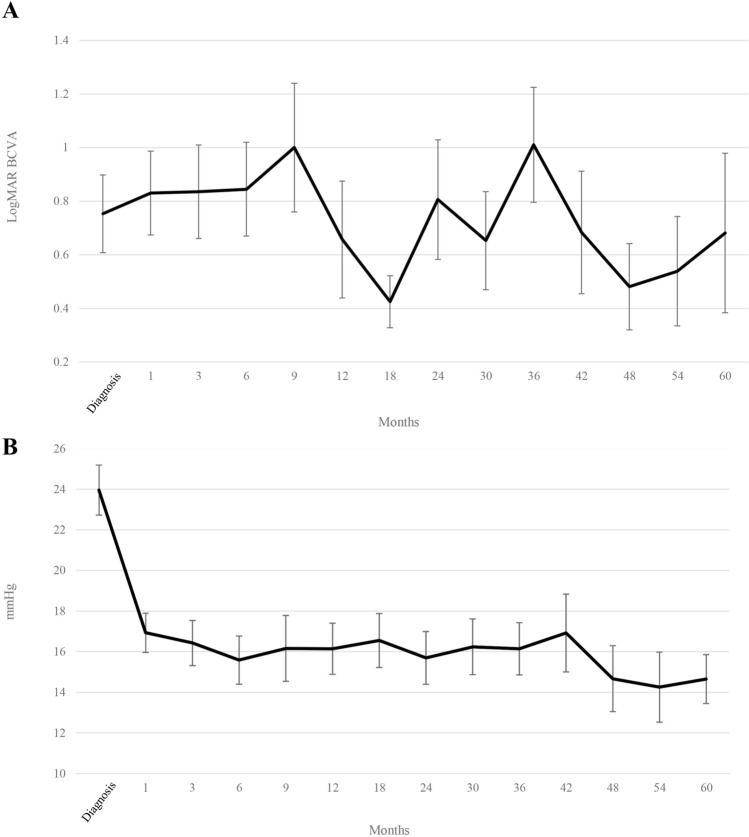
(**A**) LogMAR best-corrected visual acuity (BCVA) and (**B**) Intraocular pressure at cytomegalovirus infection diagnosis and during the follow-up period.

As for secondary outcomes, logMAR BCVAs after diagnosis are shown in Fig. 2A. The results revealed that logMAR BCVA fluctuated over time. The mean logMAR BCVAs at 1, 2, 3, 4, and 5 years after treatment were 0.7, 0.8, 1.0, 0.5, and 0.7, respectively. IOP changes after diagnosis are illustrated in Fig. 2B. The results demonstrated that mean IOP was high at diagnosis (24.0 mmHg), but less than 17 mmHg 1 month after treatment; this showed a gradually decreasing trend. Mean IOPs at, 1, 2, 3, 4, and 5 years posttreatment were 16.1, 15.7, 16.1, 14.7, and 14.6 mmHg, respectively.

ECDs were measured after treatment at the end of follow-up period. Mean ECDs in diseased and healthy eyes were 1738.3 ± 691.7 and 2466.8 ± 597.3 cells/mm^2^, respectively (*P* < 0.001). Mean corneal endothelial cell loss in the diseased eyes was 19.7% comparing to the healthy eyes. Compared with mean ECD at diagnosis, mean ECD after intravitreal ganciclovir injection did not change significantly in either diseased or healthy eyes (*P* = 0.944 and 0.615, respectively). Similarly, mean corneal endothelial cell loss did not change significantly after treatment (*P* = 0.561).

During the follow-up period, 11 patients (18.0%) received glaucoma surgery for IOP control, and 49 (80.0%) were prescribed with topical antiglaucoma agents. Five patients (8.2%) underwent corneal transplantation, whereas 19 (31.1%) underwent cataract surgery.

## Discussion

In this study, we evaluated the long-term therapeutic effects of the intravitreal injection of ganciclovir in adjunction with oral valganciclovir in patients with CMV anterior segment infection. We found that the median molecular recurrence-free survival time was 47.0 months. In addition, we identified corneal transplantation before CMV anterior segment infection diagnosis as an independent risk factor for recurrence.

Given that CMV anterior segment infection is a relatively newly emerged disease, consensus regarding the most appropriate therapy has not been reached, with the high recurrence rate after treatment cessation as a particularly challenging problem. In a large study on CMV anterior uveitis, Chee et al. reported that systemic or intravitreal ganciclovir or ganciclovir implant exhibited satisfactory response rates but also quite high recurrence rates; by contrast, ganciclovir gel had moderate response rates, but recurrence rates were relatively low^8^. Recently, topical ganciclovir has become the preferred treatment for CMV anterior segment infection, particularly for its favorable reduction of recurrence^10,13^. However, the cost-effectiveness of treating patients with long-term topical ganciclovir is questionable. Patients’ compliance, possible side effects, and emergence of resistant strains after topical ganciclovir use remain concerns. The previous result using intravitreal ganciclovir injection as a loading dose in conjunction with oral valganciclovir in six patients with CMV anterior uveitis^12^ encouraged us to continue treating our patients using the same therapeutic regimen. By extending our observation period and including additional patients, we could examine long-term outcomes, discovering that recurrence did occur but that the median 50% recurrence-free survival time was nearly 4 years. We subsequently changed the treatment strategy and started treating CMV anterior segment infection patients with topical ganciclovir. In future studies, comparison of the therapeutic effects and cost-effectiveness of these two treatment strategies is warranted.

In this study, we used molecular tests to make a definite diagnosis of recurrence. Although clinical signs such as new onset of corneal edema, elevated IOP, and anterior chamber inflammation may imply recurrence in patients with CMV anterior segment infection, distinguish inflammation from other causes may also be difficult; for instance, in post-keratoplasty patients, graft rejection and CMV endotheliitis share common features, including corneal edema, KP, and anterior chamber reaction^14,15^. Therefore, in combination with clinical signs, positive CMV DNA in the aqueous humor detected through real-time PCR during active inflammation would render a diagnosis of CMV recurrence more accurate.

Corneal decompensation and glaucomatous optic nerve changes are the major morbidities in CMV anterior segment infection. Although IOP exhibited a stabilizing trend in this study, glaucoma surgery was warranted in 11 patients (18.0%) and more than three quarters of all patients required topical antiglaucoma medication. Refractory elevated IOP occurred in patients who responded well to treatment without CMV anterior segment infection recurrence, indicating an already damaged and malfunctioning trabecular meshwork from previous CMV infection. Similarly, five patients who achieved remission after treatment underwent corneal transplantation due to severe irreversible dysfunction of the corneal endothelium, despite the clinical quiescence of CMV activity. Our findings also highlighted the necessity of early detection and treatment of CMV anterior segment infection to prevent irreversible damage to both the trabecular meshwork and corneal endothelium.

In this study, we found that corneal transplantation before the diagnosis of CMV anterior chamber infection was an independent risk factor for recurrence. Patients who have undergone corneal transplantation are usually prescribed topical corticosteroids to suppress immune reaction and prevent rejection. However, suppression of immunity may also trigger CMV reactivation in diseased eyes, resulting in the recurrence of CMV anterior segment infection^15^. Of the seven patients (11.5%) who had a corneal transplant prior to CMV anterior segment infection diagnosis, five experienced molecular recurrence in a mean time of 15.0 months after treatment and were placed on topical corticosteroids. Thus, patients with post-keratoplasty CMV anterior segment infection must be monitored closely due to a high recurrence risk. However, determining the proper use of corticosteroids and prophylactic anti-CMV drugs in such patients was beyond the scope of this study.

The major limitation of this study is its retrospective nature and the lack of control group for comparative assessment of therapeutic outcomes. Another limitation is the lack of repeated aqueous taps for polymerase chain reaction (PCR), enabling infection remission to be confirmed molecularly as well as through clinical quiescence of inflammation. Moreover, we did not keep residual aqueous samples, so further analysis such as genotypic drug resistance, which may be associated with recurrence and outcome, could not be performed. Nevertheless, having extended our previous relative short-term treatment strategy to evaluate its effect on patients with CMV anterior segment infection in the long term, we concluded that intravitreal ganciclovir injection with or without adjuvant oral valganciclovir could assist in controlling CMV anterior segment infection, with a median recurrence-free survival time of nearly 4 years and stabilized or improved BCVA, IOP, and ECD. Therefore, this therapeutic strategy seems to be effective. Because corneal transplantation before CMV anterior segment infection diagnosis was identified as an independent risk factor for molecular recurrence, a different treatment strategy could be considered for such patients. Given the satisfactory outcome of another study on topical ganciclovir treatment for patients with CMV anterior segment infection^10^, a head-to-head comparison between intravitreal ganciclovir injection with oral valganciclovir and topical ganciclovir is warranted.

## Materials and methods

### Patients

All procedures performed in studies were accordant with the ethical standards of the Helsinki declaration, and were approved by the Institutional Research Ethics Board at Chang Gung Memorial Hospital (CGMH), Taiwan. We enrolled patients diagnosed as having CMV anterior segment infection (corneal endotheliitis or anterior uveitis) at CGMH from December 2010 to March 2016. Informed consent to diagnostic aqueous tap and off-label use of intravitreal injection of ganciclovir were obtained from all patients before study participation. All patients presenting with characteristic signs of CMV anterior segment infection received a diagnostic aqueous humor tap with a 27-gauge needle during active inflammation. The specimen was sent for quantitative real-time PCR for the DNA detection of CMV, herpes simplex virus (HSV), Varicella Zoster virus (VZV), and EBV. The High Pure Viral Nucleic Acid Kit (Roche Molecular Biochemicals, Mannheim, Germany) was used to extract viral DNA from the specimen, with detection limit of 250 virus-derived DNA copies per milliliter for CMV DNA^16^. The exclusion criteria were positive HSV or VZV DNA detection in the aqueous humor, posterior segment inflammation, external eye infection, scleromalacia, or history of hypersensitivity to ganciclovir.

The medical records of patients with CMV anterior segment infection were reviewed retrospectively. We documented the demographics, medical history, and clinical findings of these patients, including age, sex, medication use and ocular surgery prior to diagnosis, CMV viral loads, BCVA, IOP, anterior chamber inflammation grading, and ECD. Anterior chamber inflammation was observed through slit-lamp microscopy and graded according to the SUN working group criteria by the same ophthalmologist (YSH). Corneal endothelial cells were evaluated through specular microscopy (CEM-530, Nidek Co., Ltd., Japan) at the corneal center in both the healthy and diseased eyes of the same patient.

During the follow-up period, we recorded IOP, BCVA, and ECD during patient visits. The presence of filtering surgery, corneal transplantation, cataract surgery, and antiglaucoma agent use were also documented. A repeated diagnostic aqueous tap was performed if elevated IOP, corneal edema, or anterior chamber inflammation recurred.

### Treatment

All patients diagnosed with CMV anterior segment infection received an IVI of ganciclovir (2 mg/0.05 mL) in the surgical room. A new vial of ganciclovir (Cymevene; F. Hoffmann–La Roche, Ltd., Basel; Switzerland) was diluted with balanced salt solution (Alcon, Fort Worth, TX, USA) in a tuberculin syringe for each patient by using an aseptic technique. After sterilization with 5% diluted povidone–iodine, an eyelid speculum was placed. Pars plana IVI of ganciclovir (2 mg/0.05 mL) in the inferotemporal quadrant was performed using a 30-gauge needle after applying topical anesthesia. After the operation, a prophylactic topical antibiotic was prescribed for 7 days.

We examined the patient postoperatively at 1 week, 1 month and at monthly intervals thereafter. The visit intervals could be shortened or lengthened according to disease activity. Prescription of adjunctive oral valganciclovir (Valcyte; F. Hoffmann–La Roche, Ltd.) depended on inflammation grading of the aqueous humor at the 1-week visit. Oral valganciclovir (900 mg) was prescribed twice a day for at least 1 month if the aqueous humor cell was equal to or more than 1 + based on the SUN working group criteria. Cessation of oral valganciclovir was determined by grading the aqueous humor cell. If the aqueous humor cell was less than 1 + , CMV infection was considered to have entered remission, and the patient was asked to maintain a monthly visit schedule without oral valganciclovir. The duration of oral valganciclovir use was recorded. Treatment success and disease remission were achieved when no cells were present in the aqueous humor.

### Outcomes measurement

The primary outcome measurement was molecular recurrence, defined as the presence of a CMV load in the aqueous humor during a repeated aqueous tap. A repeated diagnostic aqueous tap would be performed if elevated IOP, corneal edema, or anterior chamber inflammation recurred during the follow-up period. We calculated survival time as the time from the first intravitreal injection of ganciclovir to that of the first repeated aqueous tap revealing positive CMV DNA in the aqueous humor. If the PCR result for CMV DNA was negative or if the patient did not receive a repeated tap, we recorded survival time as the time from initial intravitreal injection to that of the last follow-up visit.

The secondary outcome measurements, including BCVA, IOP, and ECD. BCVA and IOP, were obtained on the day of the diagnostic aqueous tap, 1 month after intravitreal ganciclovir injection, at monthly intervals during the first year, and at half-yearly intervals for up to 5 years. ECD was measured in both healthy and diseased eyes after diagnosis and treatment. Corneal endothelial cell loss (%) was used to evaluate endothelial cell damage, and was calculated using the formula: (1-ECD in the diseased eye/ECD in the healthy eye) × 100%^10^.

In patients with positive CMV DNA in the aqueous humor after repeated tap, we changed the treatment strategy and prescribed topical ganciclovir for recurrence of CMV anterior segment infection. In patients with negative CMV DNA in the aqueous humor after repeated tap, we treated elevated IOP with anti-glaucoma topical medication or glaucoma surgery. Corneal transplantation was performed in eyes with severe corneal edema.

### Statistical analysis

Categorical variates are presented with number with percentage, whereas continuous variates are presented as means ± standard error. The longitudinal change of IOP and BCVA was demonstrated as means ± standard error of the mean in the figures. Risk factors of molecular recurrence were estimated using univariate and multivariate Cox regression analysis. BCVA values were transformed from the decimal notation to logarithm of the minimum angle of resolution (logMAR). Kaplan–Meier survival analysis was used to estimate the probability of positive CMV DNA recurrence over the follow-up period. Comparison of ECD was performed with independent *t* test. A *P* value of < 0.05 was considered statistically significant.

## Data Availability

The data used for the current study will be available from the corresponding author upon reasonable request.
